# Seasonal forecasting using the GenCast probabilistic machine learning model

**DOI:** 10.1007/s00382-026-08077-4

**Published:** 2026-03-16

**Authors:** Bobby Antonio, Kristian Strommen, Hannah M. Christensen

**Affiliations:** 1 Atmospheric, Oceanic and Planetary Physics, University of Oxford, Sherrington Road, Oxford, OX1 3PU UK; 2 European Centre for Medium-Range Weather Forecasts, Shinfield Rd, Reading, RG2 9AX UK

**Keywords:** Seasonal forecasting, Machine learning, Numerical weather prediction

## Abstract

Machine-learnt weather prediction (MLWP) models are now well established as being competitive with conventional numerical weather prediction (NWP) models in the medium range. However, there is still much uncertainty as to how this performance extends to longer timescales, where interactions with slower components of the earth system become important. We take GenCast, a state-of-the-art probabilistic MLWP model, and apply it to the task of seasonal forecasting with prescribed sea surface temperature (SST), by providing anomalies persisted over climatology (GenCast-Persisted) or forcing with observed SSTs (GenCast-Forced). The forecasts are compared to the European Centre for Medium-Range Weather Forecasts seasonal forecasting system, SEAS5. Our results indicate that, despite being trained at short timescales, GenCast-Persisted produces much of the correct precipitation patterns in response to El Niño and La Niña events, with several erroneous patterns in GenCast-Persisted corrected with GenCast-Forced. The uncertainty in precipitation response, as represented by the ensemble, compares favourably to SEAS5. Whilst SEAS5 achieves superior skill in the tropics for 2-metre temperature and mean sea level pressure (MSLP), GenCast-Persisted achieves higher skill in some areas in higher latitudes, including mountainous areas, with notable improvements for MSLP in particular; this is reflected in a slightly higher correlation with the observed NAO index. Reliability diagrams indicate that GenCast-Persisted has little skill relative to climatology, whilst GenCast-Forced produces forecasts with reliability comparable to SEAS5. These results provide an indication of the potential of MLWP models similar to GenCast for the ‘full’ seasonal forecasting problem, where the atmospheric model is coupled to ocean, land and cryosphere models.

**Supplementary Information:**

The online version contains supplementary material available at 10.1007/s00382-026-08077-4.

## Introduction

Recent years have seen a proliferation of machine-learnt weather prediction (MLWP) models that are competitive with conventional physics-based models at medium-range weather forecasting, for both deterministic (Lam et al. 2023; Bi et al. 2023; Allen et al. 2025) and probabilistic forecasts (Price et al. 2025; Lang et al. 2024). So far, these models have focused mainly on short- to medium-range weather forecasts. A natural question to ask is whether these models could be used to forecast to longer horizons, specifically out to seasonal timescales.

Since current MLWP models do not have an interactive ocean, seasonal forecast experiments using these models must currently prescribe an evolving ocean state as a boundary condition. In this context, a seasonal forecast experiment primarily tests the ability of the MLWP atmosphere to respond correctly to the changing ocean state. There are several benefits of such an experiment. Firstly there is the practical aspect of evaluating how skilful these models are at seasonal timescales; if successful, then MLWP forecasts would offer a means to efficiently generate large seasonal forecast ensembles and potentially produce more skilful and reliable forecasts. Rolling out to longer timescales also serves as a useful test of the physical realism of models, and how well they generalise to tasks they are not trained on. Seasonal forecasting in particular is a test of how well the MLWP model has learned to respond to other Earth system components such as the ocean. Such applications to different tasks can build trust in the output of these models, and provide insight into how general purpose the models can be. It can also reveal undesirable behaviours of MLWP models that are not apparent at shorter timescales. For example, these experiments allow an assessment of how stable the models are at long timescales, which is important since several are known to become unstable and produce unrealistic values outside of the 14-day horizon (e.g. Karlbauer et al. (2024)). Finally, it is of direct scientific interest to understand to what extent accurately simulating short timescale weather phenomenon automatically allows longer timescale variability to be accurately simulated as well; such understanding has direct implications for, e.g., the ‘seamless prediction’ framework (Palmer et al. 2008; Christensen and Berner 2019), wherein one tries to use information about short-term weather forecasts to constrain climate projections in models.

In order to perform forecasts beyond the medium range, there are two main approaches to consider. In the ‘direct’ approach, a machine learning model is trained to directly predict the forecast variables at the lead times of interest. There are several studies that apply this approach to subseasonal to seasonal (S2S) forecasting (up to around 6 weeks lead time, Delaunay and Christensen 2022; Nguyen et al. 2023; Liu et al. 2025) and seasonal forecasting (Pinheiro and Ouarda 2025). As an alternative to forecasting the atmosphere, others have demonstrated how machine learning models can predict key drivers of seasonal variability such as the El Niño/Southern Oscillation (ENSO) index (Ham et al. 2019; Parthipan et al. 2025).

Alternatively, we can adopt an ‘autoregressive’ approach, by which we mean a model that makes predictions at a daily or sub-daily level, and is rolled out to seasonal timescales. In this approach it is hoped that a MLWP model trained at relatively short timescales will learn the correct physical interactions in order to create the correct behaviour at longer timescales. There are several studies applying this approach for S2S timescales (Chen et al. 2024; Li et al. 2025; Chen et al. 2024; Weyn et al. 2024; Ling et al. 2024; Zhou et al. 2025). However, to our knowledge, this autoregressive approach has been tested on seasonal timescales in only two works: Kent et al. (2025) use the ACE2 model (Watt-Meyer et al. (2025)) to perform seasonal forecasts, with a model that is forced with SST and sea ice cover anomalies, where ensembles are created using a lagged ensemble approach. Zhang et al. (2025) perform seasonal hindcasts with NeuralGCM (Kochkov et al. 2024), similarly using persisted SST and sea ice anomalies, with a focus on forecasting tropical cyclone activity, and creating ensembles using initial condition perturbations. We note that both ACE2 and NeuralGCM were designed with climate applications in mind.

In this work, we are are interested in further exploring the autoregressive approach applied to seasonal forecasting, since it provides an interesting test of the kind of physical relationships that MLWP models can learn having being trained at short timescales. It is also a useful precursor to assess how different models could extend to climate timescales. We use GenCast (Price et al. 2025), a recently developed probabilistic model that achieves state-of-the-art skill in the medium range, and explore how well it performs at the task of seasonal forecasting over a four month period with prescribed sea surface temperatures. Our setup mirrors that of Kent et al. (2025) and Zhang et al. (2025) in that persisted SST anomalies are used as boundary condition, although we also consider a forced setup where ERA5 SSTs are provided, in order to assess where forecast skill is limited by factors beyond the ocean representation. Aside from being the first application of GenCast to forecasting beyond the medium-range, our work complements existing studies in several ways. Firstly, GenCast is a probabilistic model, which in theory can learn to directly predict the correct conditional probability distribution. We may therefore expect it to produce a more reliable ensemble compared to initial condition or lagged ensembles. GenCast was also designed specifically for the medium-range, unlike NeuralGCM and ACE2. Evaluating the model on seasonal timescales may reveal biases in GenCast that are not apparent at short lead times, and evaluates whether a model designed purely for the medium-range can possibly generalise to longer timescales. Finally, given the relatively small number of studies for seasonal prediction using autoregressive models, it is a useful additional case study, to explore any potential benefits or disadvantages of using a different model.

## Methods

### Machine learning model

GenCast makes predictions at 12hr time steps, for 6 surface variables, and 6 variables at 13 pressure levels. We use the $1^{\circ }$ model since the GPU available to us was not large enough to fit the $0.25^{\circ }$ version. GenCast receives no inputs related to the land surface (e.g. soil moisture) and, unlike ACE2 and NeuralGCM, does not take information about sea ice as an input. Each GenCast forecast is initialised on the 1st November, and rolled out until the end of the following February. We initialise the forecasts on years 1994-2024; this is to incorporate as much data as possible that is completely unseen by GenCast (2019-2024), as well as incorporating years with a range of different conditions. Note that, even though the years 1994-2018 are within the training period for GenCast, by rolling the forecast out autoregressively to seasonal timescales, we are still exposing the model to inputs it has not seen before. These years can therefore also be considered out-of-sample for this experiment. In order to verify that this is a reasonable assumption, globally averaged CRPS scores were calculated for each of the initialisation years (Fig. S1 in Supplementary Information), which showed no obvious degradation in CRPS scores over the out-of-sample period compared to the training period.

GenCast is run with two different ocean boundary conditions. The first setup, GenCast-Persisted, persists the ERA5 anomalies at 1st November on top of the SST climatology for the duration of the forecast, similarly to the approach in Kent et al. (2025) and Zhang et al. (2025), based on the approach in Zhao et al. 2010. This setup is closest to a forecast setup, where the real sea surface temperatures are not known in advance. The climatology used for this experiment is the daily SST climatology calculated over the ERA5 data from 1^st^ January 1979–12th December 2018. The second setup, GenCast-Forced, uses ERA5 sea surface temperature as input to GenCast. This serves as a useful indicator of where skill or reliability might be improved by a more accurate representation of the ocean.

### Data

The ERA5 reanalysis dataset (Hersbach et al. 2020) is used as the ’ground truth’ for 2-metre temperature and mean sea level pressure, since this is the dataset GenCast is originally trained on. For precipitation, we compare with the Global Precipitation Climatology Project (GPCP) version 3.2 monthly dataset (Behrangi et al. 2024); note that this dataset only has temporal coverage up until December 2023, so precipitation verification is performed over initialisations on 1^st^ November from 1994-2022. Climatology for each dataset is estimated by averaging the forecasts and observations by month over the 30-year period.

As a baseline forecast, we use the European Centre for Medium Range Weather Forecasts (ECMWF) SEAS5 forecasts (Johnson et al. 2019). For both SEAS5 and the GenCast experiments we use 20 ensemble members. Data is aggregated to give an average value for the boreal winter (December-February), and is detrended when calculating the anomaly correlation coefficient and reliability diagrams to remove the climate change signal. Subregions are chosen to explore the precipitation distribution response to El Niño / La Niña in Sect. 3.1.1, taken from the regions in Davey et al. (2014) for which there is a wetting or drying signal over December-February for both types of events. The subregions (using the same naming conventions as in Davey et al. (2014)) are Indonesia ([10^o^S-5^o^N, 100^o^-130^o^E]), SSAfrica ([28^o^-18^o^S, 18^o^-33^o^E]) and MexUSA ([30^o^-35^o^N, 120^o^-90^o^W]). Averages are taken over land points only, with the exception of Indonesia which includes land and sea points.

### NAO index

We calculate the North Atlantic oscillation (NAO) index as the difference in mean sea level pressure for a region around the Azores ([28-20^o^W, 36-40^o^ N]) and around Iceland ([25-16^o^ W, 63-70^o^ N]), following Dunstone et al. (2016). The NAO series for each forecast is centred by subtracting the mean NAO value for that series over the 30-year period. Each series is then normalised by dividing by the standard deviation of the NAO index calculated on ERA5 data.

### Tests of significance

In order to test where anomaly correlations $r_{a}$ are significantly greater than 0, we use a one-sided t-test of the test statistic $r_{a} ( n-2)^{1/2} / (1-r_{a}^2)^{1/2}$ (Von Storch and Zwiers 1999), where *n* is the number of years used to calculate the result. In order to test where there is a significant difference between the anomaly correlation of two different forecasts, we follow the approach outlined in Siegert et al. (2017), which accounts for the fact that the two forecasts are themselves correlated. All significance results are reported at the 95% confidence level.

### Reliability diagrams

To calculate the reliability diagrams, the forecasts and observations are separately detrended in order to remove any climate change signal. Terciles are then calculated for each grid cell individually, allowing a calculation of probabilities for each grid cell separately.

## Results

### Process-based analysis

In this subsection, we evaluate GenCast’s ability to forecast key processes in the Earth system. We begin by looking at El Niño-Southern Oscillation (ENSO) via a case study of two years with strong El Niño or La Niña conditions. Since ENSO is one of the key ocean-related drivers of atmospheric variability (McPhaden et al. 2006), it is important that MLWP models can model the atmospheric response — both mean and variability — correctly. We then look at how well GenCast can predict the North Atlantic Oscillation, which is an important driver of weather and climate variability in Eurasia and North America (Hurrell et al. 2003)

#### El Niño and La Niña case studies

**Fig. 1:**
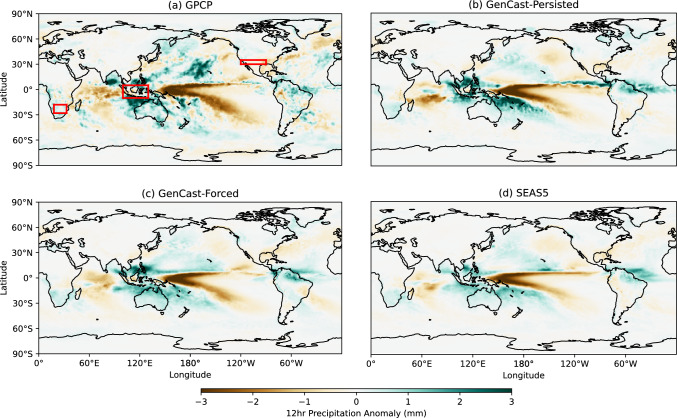
Seasonal 12hr precipitation anomalies for December 2010–February 2011 (Strong La Niña). Red boxes indicate the subregions used in Fig. 3

**Fig. 2:**
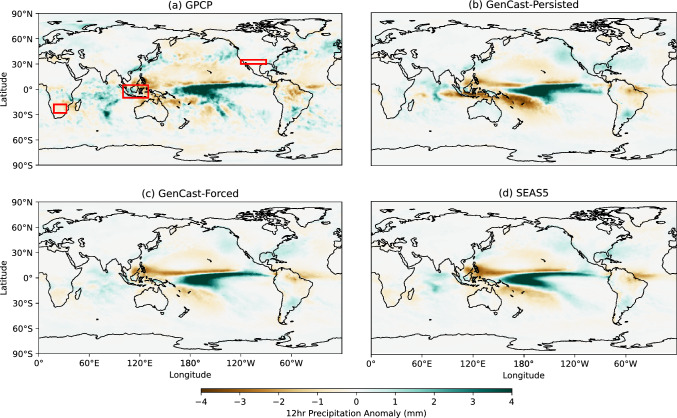
As for Fig. 1, but for December 2015–February 2016 (Strong El Niño)

In order to gain insight into how GenCast responds to sea surface temperature anomalies, we investigate precipitation forecasts produced when initialised in a year with strong El Niño or La Niña conditions. The periods chosen are December 2010–February 2011 (strong La Niña conditions) and December 2015–February 2016 (strong El Niño conditions), selected as they are the years with strongest ENSO signal. Whilst these periods are within GenCast’s training period, this still represents an out-of-sample experiment since we are rolling GenCast autoregressively out to seasonal timescales far beyond the model’s short training timescales. The resulting ensemble mean 12hr precipitation anomalies for December-February are shown in Figs. 1 and 2, using 20 ensemble members for both SEAS5 and GenCast, and compared with GPCP.

For the 2010-2011 La-Niña forecast anomalies shown in Fig. 1, we can see that both GenCast-Persisted and GenCast-Forced (panels b and c) produce a distinct pattern of drying over the tropical Pacific and wetting over the maritime continent, in agreement with GPCP and SEAS5 (panels a and d). Some difference can be seen between GenCast-Persisted and GPCP; e.g. there are additional wetting signals in the Hudson Bay, eastern Pacific, and Indian Ocean, although these are removed with GenCast-Forced. Both GenCast forecasts also capture the correct pattern of drying and moistening associated with La Niña away from the Pacific basin, for example over South America, and over central and southern Africa.

For the 2015-2016 El Niño forecast anomalies in Fig. 2, we broadly see an agreement in the pattern of wetting and drying over the tropical Pacific and maritime continent for all models. The shape of the wetting signal in the tropical Pacific and tropical Atlantic for GenCast-Persisted (panel b) is however quite different to observations, and the model predicts too much drying over the maritime continent; however, these artefacts are removed when moving to GenCast-Forced (panel c). Both GenCast models appear to show a slightly more accurate representation of the wetting and drying pattern in the northern Atlantic. Both GenCast models also seem to produce a correct rainfall response in South America, with drying over Brazil and wetting over Argentina, in agreement with the observations. However, in this instance SEAS5 predicts a wetting signal over the Amazon region and southern Brazil that is not consistent with observations. Similarly, over South Africa all models show a drying response consistent with observations.

**Fig. 3:**
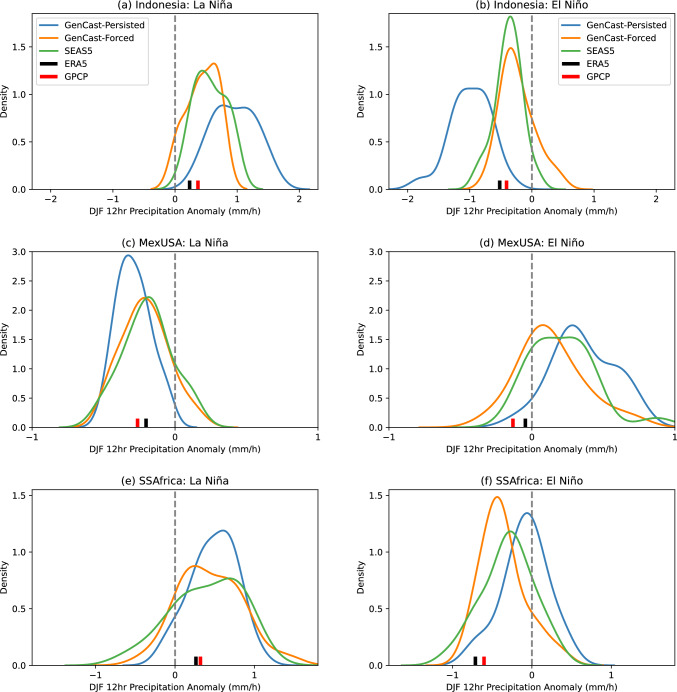
Kernel density estimate for the distribution of DJF 12hr precipitation anomalies, averaged over each of the subregions shown in Figs. 1 and 2 (rows), and for each of the La Niña and El Niño case studies (columns). In each plot the ERA5 value is shown in black

Apart from the ensemble mean prediction, it is also important to check that the ensemble distribution is reasonable compared to the physical forecast and observations. In Fig. 3 we show the distribution across the ensemble of 12 hour precipitation anomalies averaged over several regions, chosen from Davey et al. (2014) as areas with a notable response to El Niño and La Niña in December-February (see Sect. 2.2). Overall we can see that GenCast-Persisted and GenCast-Forced produce distributions of a similar spread and mean value to SEAS5, with clear shifts across the 0 anomaly line between the La Niña and El Niño years, and such that the ERA5 data point (black line) lies within each of the distributions. For Indonesia in panels a and b (wetter/drier during La Niña/El Niño), we can see that the the bulk of the forecast distributions fall on the expected side, with GenCast-Persisted having the greatest variation between El Niño and La Niña, whilst the SEAS5 and GenCast-Forced distributions are fairly similar. For MexUSA in panels c and d (drier/wetter during La Niña/El Niño), all models show a shift to increased precipitation moving from La Niña to El Niño conditions, with GenCast-Persisted showing a particularly pronounced drying signal during the La Niña year. For SSAfrica in panels e and f (wetter/drier during La Niña/El Niño), all forecasts show clear drying and wetting signals, with similar shaped distributions, although GenCast-Forced showing a particularly pronounced drying signal during the El Niño year.

In summary, GenCast is able to capture the observed response to these two strong ENSO events, indicating that it has learned to correctly replicate some of the atmospheric response to sea surface temperature, despite this interaction not being a dominant driver in skill at the timescales it was trained at. This is also reflected in the change in distribution of precipitation averaged over several subregions; there is a clear shift in all of the forecast distributions that reflects the expected wetting and drying behaviour for each subregion in response to the ENSO conditions, and the distributions of both GenCast-Persisted and GenCast-Forced show similar spread and mean to the SEAS5 distribution.

#### NAO prediction

**Fig. 4:**
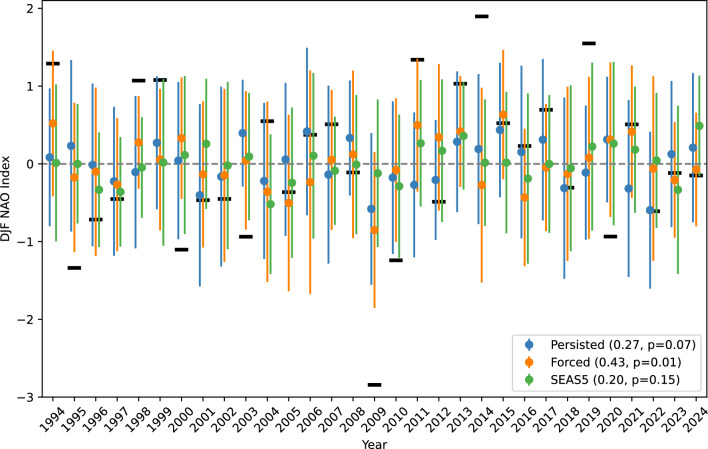
NAO Index calculated from the mean sea level pressure predictions of GenCast, aggregated by season. Error bars indicate the spread of the ensemble members. Numbers in brackets are the Pearson correlation of each time series with ERA5, including an estimated p-value (one-sided t-test)

In this section we evaluate how well GenCast predicts the North Atlantic Oscillation (NAO). The predictions of the NAO index are shown in Fig. 4, where each time series has been centred by subtracting its mean over the 30 year period, and normalised by dividing by the standard deviation of the index calculated on ERA5 data.

SEAS5 systematically underestimates the variability of NAO values compared to observations, with a correlation of just 0.20 (not significant at the 5% level). The underestimation of the magnitude of NAO variability is related to the so-called ‘signal-to-noise’ problem, a problem shared by all physical models capable of performing skillful NAO forecasts (Scaife and Smith 2018; Johnson et al. 2019). Interestingly, the MLWP forecasts share the same issue, consistent with the result of Watt-Meyer et al. (2025). With regards to the skill, GenCast-Persisted obtains a slightly higher correlation of 0.27 than SEAS5 does over this time period, although also not significant at the 5% level. There is a reasonable improvement realised with GenCast-Forced (significant correlation of 0.43), consistent with the increased skill seen for the MSLP over the North Atlantic in Sect. 3.2.1. It is interesting to note that for the extremely negative NAO event of 2009/2010, both GenCast models also show an unusually negative NAO relative to the rest of the series, particularly GenCast-Forced, whilst the SEAS5 prediction is close to zero.

We emphasise that there is a crucial caveat to the above results, namely a strong sensitivity to ensemble size. When using all 51 available members, the NAO correlation achieved by SEAS5 is higher, at 0.43 (Johnson et al. 2019). Such sensitivity of NAO correlations to ensemble size are well known, and given multiple 20-member ensembles from the same model one can expect the correlations obtained to vary randomly by around 0.2 or more: see, e.g., Dunstone et al. (2016), their Figure 2c. This same figure also highlights that skill with 50 or more members is expected to be notably higher than with 20 members, and thus our 20-member ensemble will likely underestimate the real skill, consistent with the higher correlation of 0.43 reported for SEAS5 in Johnson et al. (2019). With only 20 members it is therefore difficule to make a strong statement about the relative performance of GenCast-Persisted, GenCast-Forced and SEAS5 when it comes to the NAO. The main takeaway here should therefore probably just be that GenCast has a comparable performance to SEAS5.

### Assessment of skill

#### Anomaly correlation

In this section we evaluate the skill of GenCast in predicting seasonal 2-metre temperature (T2m) and mean sea level pressure (MSLP), using the anomaly correlation coefficient (ACC). Before calculating the correlation, all anomalies at each grid cell are detrended to remove any climate change signal.

**Fig. 5:**
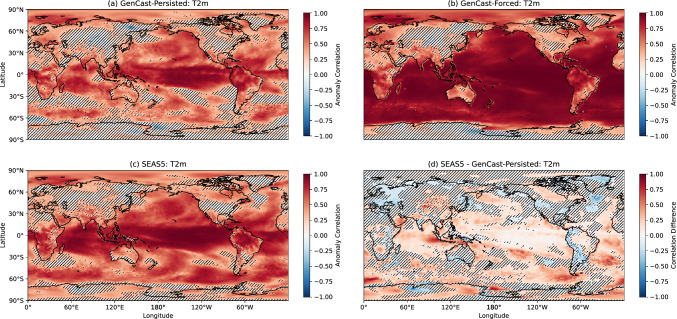
Anomaly correlation coefficient (ACC) for DJF 2-metre temperature, for forecasts initialised on 1^st^ November 1994-2024. Higher ACC indicates more skill. Hatching indicates where correlations or correlation differences are significant at the 95% level (see Sect. 2.4)

The ACC results for T2m are shown in Fig. 5. Panels a and c show that there are similar patterns of skill for GenCast-Persisted and SEAS5, with both generally achieving higher correlation in the tropics. The correlations close to 1 for GenCast-Forced over the ocean (panel b) show that GenCast is using the SST input appropriately to set the 2-metre temperature, whilst the low skill over much of the land points highlights the need for more land surface information in GenCast’s inputs. Figure. 5 (d) confirms that there is no significant difference between the skill of SEAS5 and GenCast-Persisted over large parts of the extratropics, though SEAS5 is significantly more skilful over tropical ocean regions, particularly around the maritime continent, as well as the northern and southern Pacific. In contrast, GenCast-Persisted outperforms SEAS5 over some mountainous regions including the Andes and the Himalayas. It is also interesting to note that GenCast-Persisted shows a slight improvement in the North-West Atlantic, a feature attributed to how SEAS5 captures the variability of the North Atlantic subpolar gyre (Johnson et al. 2019). There also appear to be slight improvements over land in eastern Europe, western Russia, and northern Greenland, which is perhaps surprising given GenCast does not model land processes.

Since GenCast does not does not receive any information about the sea ice, such as ice concentration or temperature, we might expect significant differences over areas of high sea ice concentration. Whilst SEAS5 does seem to perform significantly better over some of the Ross and Lazarev seas, GenCast-Persisted generally shows comparable skill in other regions such as the Weddell Sea and the Artic ocean. The area-weighted ACC results for T2m for different regions are shown in Table 1. From this we can see that overall GenCast-Persisted performs comparably to SEAS5 in the northern extratropics, with larger differences seen in the tropics and southern extratropics (differences in correlation of 0.13 and 0.14 respectively).

**Fig. 6:**
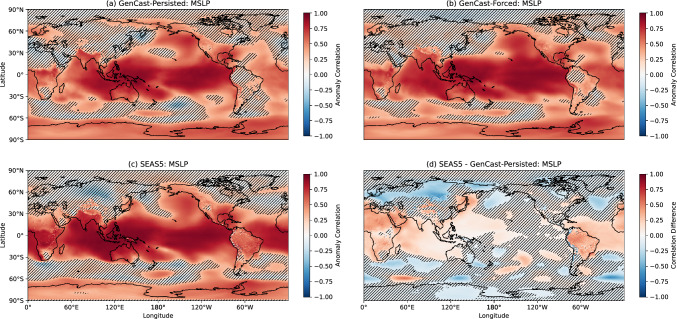
As for Fig. 5 but for mean sea level pressure

The ACC results for MSLP are shown in Fig. 6. Again all forecasts show similar patterns of skill, with SEAS5 significantly outperforming GenCast-Persisted over much of Africa, South America, around Japan, the Indian Ocean, western tropical Pacific and tropical Atlantic (panel d). There are, however, several areas in the midlatitudes where GenCast-Persisted appears to improve upon SEAS5, such as over Siberia, the northern Pacific, the North Atlantic ocean and several areas in the Southern Ocean. There is a less pronounced difference between GenCast-Persisted and GenCast-Forced for MSLP, suggesting that an accurate representation of the ocean is not sufficient to achieve much higher skill for this field, or perhaps reflecting the importance of atmosphere-ocean coupling in these regions. The area-weighted ACC results for MSLP aggregated by region are shown in Table 1. From this we can see that differences in skill between GenCast-Persisted and SEAS5 are concentrated in the tropics, with the two models performing similarly in the extratropics.

**Fig. 7:**
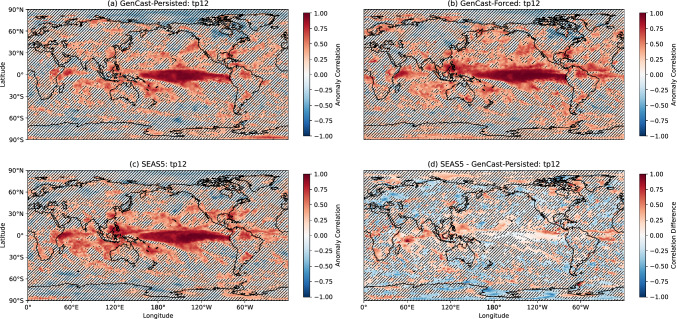
As for Fig. 5 but for 12hr precipitation, and using GPCP data in the range 1994-2022

Results for 12hr precipitation are shown in Fig. 7 (a)-(d). GenCast-Persisted (panel a) shows correlation values close to 1 over the tropical Pacific, with other notable areas of skill such as East Africa, South America, around the Gulf of Mexico, eastern China, and around the maritime continent. The most prominent differences for GenCast-Forced (panel b) are the improvements in skill over the tropical Atlantic and Indian Ocean, with the pattern of skill being otherwise quite similar, and similar patterns of skill seen for SEAS5 (panel c) and GenCast-Forced (panel b). The difference in skill between GenCast-Persisted and SEAS5 (panel d) is quite noisy, but there appear to be some areas of higher skill for SEAS5 in the tropics, particularly the Indian Ocean and tropical Atlantic. This is reflected in area-weighted ACC results for 12-hour precipitation in Table 1. From this we can see a difference in correlation of $\sim 0.1$ between GenCast-Persisted and SEAS5 in the tropics, whilst the two models have similar skill in the extratropics. GenCast-Forced achieves similar skill to SEAS5 in the tropics, suggesting that a more realistic representation of the ocean would improve GenCast’s skill in this area. The skill of GenCast-Forced in the tropics is presumably in part driven by its ability to simulate the ENSO response accurately as seen in Sect. 3.1.1. In the extratropics, GenCast-Forced seems to improve upon GenCast-Persisted mainly in the southern hemisphere.

**Table 1:** Area-weighted average anomaly correlation coefficient results by region.

	T2m Tropics	T2m Northern Extratropics	T2m Southern Extratropics
GenCast-Persisted	0.63	0.37	0.49
GenCast-Forced	0.85	0.56	0.83
SEAS5	0.76	0.40	0.63
	MSLP Tropics	MSLP Northern Extratropics	MSLP Southern Extratropics
GenCast-Persisted	0.62	0.28	0.51
GenCast-Forced	0.69	0.30	0.56
SEAS5	0.77	0.28	0.57
	12hr Precip Tropics	12hr Precip Northern Extratropics	12hr Precip Southern Extratropics
GenCast-Persisted	0.30	0.14	0.22
GenCast-Forced	0.40	0.17	0.29
SEAS5	0.39	0.14	0.27

#### Reliability of the ensemble

**Fig. 8:**
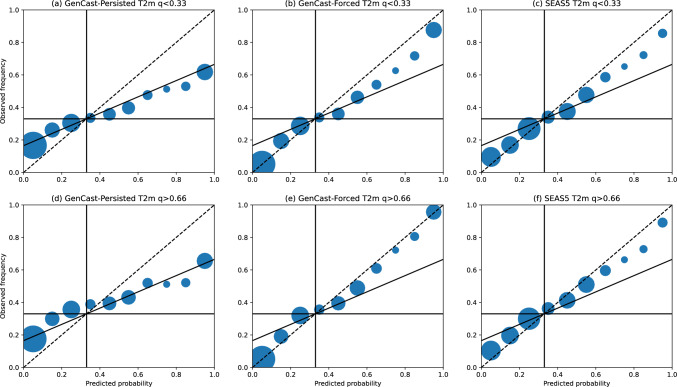
Reliability for each model, where the forecast objective is to predict the probability of 2-metre temperature anomalies in December-February being below the lower tercile (panels a-c), or above the upper tercile (panels d-f). The black dashed line in each plot indicates the line a perfectly reliable forecast would lie along. Circle sizes indicate the number of examples within each probability bin, and circles are shown at the centres of each probability bin

**Fig. 9:**
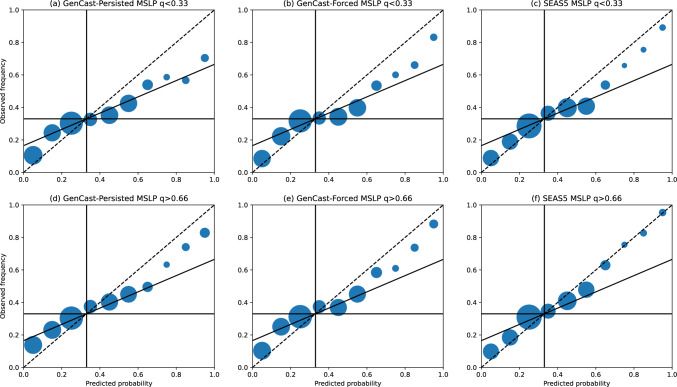
As for Fig. 8, except for mean sea level pressure anomalies

**Fig. 10:**
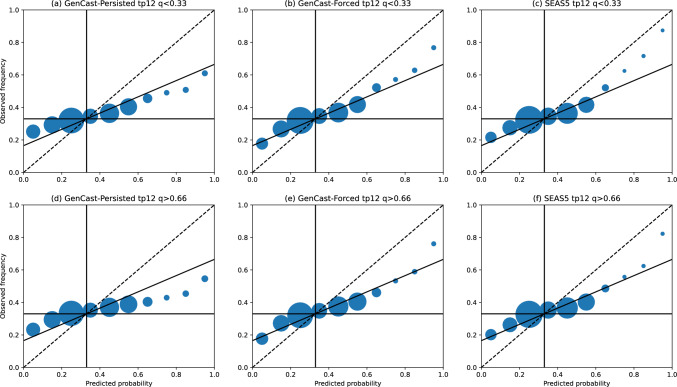
As for Fig. 8, except for 12-hr precipitation anomalies

For any probabilistic forecasting system it is important that the forecast probabilities are good indicators of how likely an outcome is, in order for the forecast to have value to decision makers. Whilst the skill of a probabilistic forecast can be summarised by one of many forecast skill metrics, a reliability diagram provides a fuller insight into the joint probability distribution of the forecast and observations for a particular binary event of interest (Wilks 2011; Hsu and Murphy 1986). Reliability diagrams for T2m, 12-hr precipitation, and MSLP anomalies are shown in Figs. 8-10, where in each plot we compare the forecast probability and observed frequencies of the seasonal value being below the lower tercile (panels a-c) or above the upper tercile (d-f). In each plot, the dashed line indicates the line of perfect reliability, where forecast probabilities correspond exactly to observed probabilities, whilst the horizontal black line indicates the line of ‘resolution’, where observed frequencies equal the climatological event frequency for all forecast probabilities. The intermediate black line indicates where the resolution equals reliability (Wilks -2011), below which the forecast does not have superior skill to climatology.

For T2m, the reliability diagrams for GenCast-Persisted, in Fig. 8 (a) and (d), show that the forecast lies closely to the no-skill line, therefore showing that the forecasts are not more skillful than climatology. In contrast, SEAS5, shown in panels (c) and (f), gives forecast probabilities more closely aligned with the observed frequencies for both events. For GenCast-Forced, shown in panels (b) and (e), we can see that the reliability becomes more aligned with the dashed line, particularly for the upper tercile events; this indicates that GenCast combined with a realistic representation of the ocean variability may be enough to produce a well-calibrated probability distribution of these events.

For MSLP in Fig. 9, GenCast-Persisted shows some skill for upper tercile events at forecast probabilities above 0.6 (panel d), but no skill for lower tercile events. GenCast-Forced improves on this a little in both cases (panels b and e), but does not reach the same skill level as SEAS5 (panels c and f), particularly for the upper tercile events.

For 12-hour precipitation in Fig. 10, again we see that GenCast-Persisted (panels a and d), lies along or below the no-skill line, with skill particularly low for upper tercile events. Whilst SEAS5 and GenCast-Forced seem to improve reliability for events with forecast probabilities above 0.6, both forecasts are still concentrated along the no-skill line. This indicates that, whilst there may be some improvements for certain events, realistic ocean input is not sufficient to improve the reliability of precipitation forecasts.

Overall, whilst GenCast-Persisted does not achieve the same reliability as SEAS5, and is often of similar skill to climatology, GenCast-Forced achieves similar reliability to SEAS5. This indicates that the uncertainty quantification learned by GenCast over short timescales is realistic when conditioned on an accurate ocean representation, even when rolled out to timescales beyond which the model was trained on.

## Model drift and trends

An important consideration for seasonal forecasts is the extent to which the models change over time. In this section we briefly examine the drift of the GenCast and SEAS5 forecasts, as well as the trend in 2-metre temperature.

**Fig. 11:**
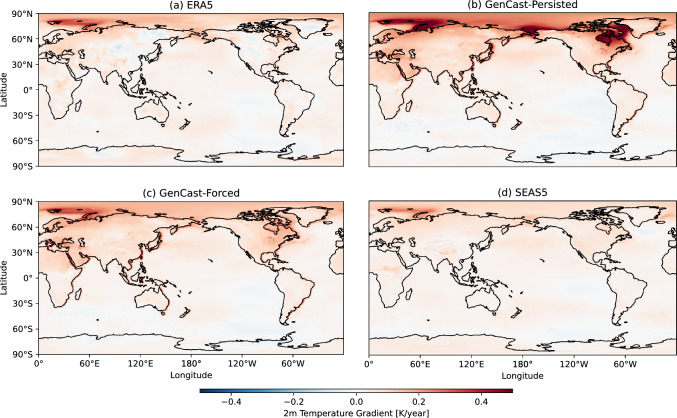
Trend in DJF 2-metre temperature for each seasonal forecast

To begin with, we explore the long-term trend of the seasonal predictions of 2-metre temperature. GenCast-Persisted and GenCast-Forced are both given initial conditions and SSTs that provide the relevant climate signal, so we would expect that these models will in some way reflect the observed warming signal in ERA5. However, it is not clear if they will mirror it exactly or introduce some artefacts, by for example regressing to the mean. Maps of trends of DJF 2-metre temperatures are shown in Fig. 11. GenCast-Persisted shows an anomalous increase in warming over the northern polar regions; these are areas where sea-ice variability is high, so this is likely to be because the anomalies used as input to GenCast-Persisted do not accurately reflect sea ice changes. GenCast-Forced is more closely aligned with ERA5 and SEAS5, although with some larger trends over North America, and many coastal regions in Asia and Europe. Note that the ACC results in Sect. 3.2.1 are linearly detrended, so the linear climate signal does not contribute to the ACC skill.

**Fig. 12:**
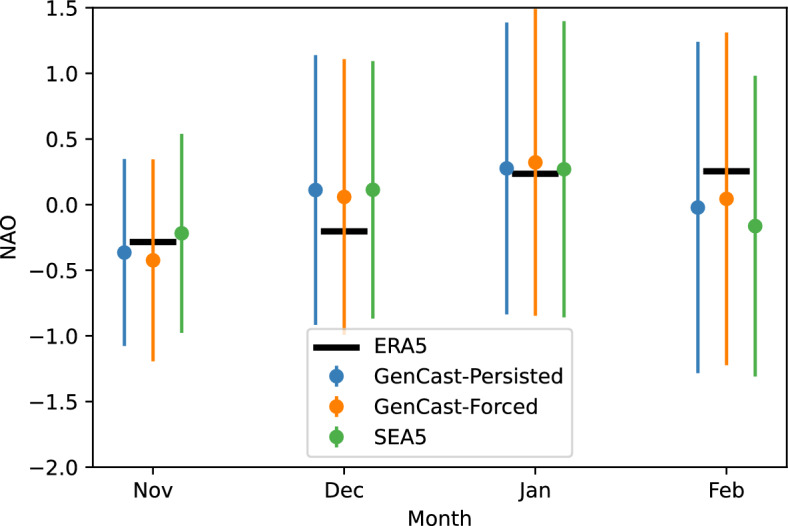
NAO index averaged over all years for each month separately

To examine climatological drift within the season, we calculate the average NAO index for each month, including November (Fig. 12). Since SSTs are prescribed, we expect that drift would be confined to the first month, as this is the approximate time taken for the atmosphere to spin up. We can see that, although there could be some drift in February away from ERA5, any differences are well within the spread of the models, and so there is no obvious indication of drift for the NAO. We this conclude that model drift and trends in the GenCast forecasts considered here are entirely comparable to those of SEAS5.

Additional plots showing the mean state bias, change in skill aggregated by forecast month and initialisation year are also provided in the Supplementary Information, Sect. S2.

## Discussion

In this work we have demonstrated the first application of GenCast to seasonal forecasting, far beyond the timescale it was trained at, by running the model for 4 months with prescribed sea surface temperature (SST) boundary conditions: In the first setup, GenCast receives persisted SST anomalies (GenCast-Persisted), whilst in the second setup GenCast receives SSTs from ERA5 (GenCast-Forced). Whilst these are not full seasonal forecasts, as they lack an interactive ocean, they provide a test into how well GenCast has learned to model long term physical processes having being trained on single timestep predictions and optimised for the medium-range. The model produces a 4-month forecast at 1^o^ resolution in around half an hour on a single A100 GPU, compared to around 2-3 hours on 50 cores for the IFS using the T255L62 grid ($\sim 78\text {km}$ resolution, Mogensen and Keeley (2012)); whilst not as fast as some deterministic MLWP models, it still offers the potential to achieve higher ensembles much more efficiently than SEAS5.

An evaluation of precipitation for two years with strong El Niño / La Niño SST warming patterns shows that GenCast is able to capture the systematic patterns of wetting and drying appropriately. An investigation of the distribution of 12hr DJF precipitation anomalies averaged over three particular subregions also demonstrates distinct shifts in distribution in response to the ENSO conditions, with distributions that align well with SEAS5 in terms of mean value and spread.

Anomaly correlations of 2-metre temperature calculated over the full 30-year dataset reveal that, whilst SEAS5 tends to achieve higher skill in many areas, particularly in the tropics, several areas in the extratropics and some mountainous regions appear to exhibit some improvement in skill. Whilst there are areas of high sea ice concentration, such as the Ross Sea, for which SEAS5 achieves significantly higher skill, GenCast-Persisted achieves comparable skill in the Arctic Ocean and Weddell Sea, which is perhaps surprising since it receives no information about the sea ice concentration or temperature. GenCast-Forced achieves anomaly correlations close to 1 over many of the ocean points, which confirms that SST input is being used appropriately to inform the 2-metre temperature. Over land points, however, there are still many areas where GenCast-Forced achieves low correlation, highlighting the need for more land surface information to be included in the model inputs.

Anomaly correlations of mean sea level pressure (MSLP) show that SEAS5 achieves superior skill in the tropics, although in the midlatitudes there are areas such as Siberia and the northern Atlantic for which GenCast-Persisted achieves higher skill. This is reflected in forecasts of the North Atlantic Oscillation (NAO) index, for which GenCast-Persisted achieves higher correlation with the NAO index calculated using ERA5, although neither GenCast-Persisted or SEAS5 produce p-values that indicate correlation significantly greater than 0. Differences in skill between GenCast-Persisted and GenCast-Forced for MSLP are relatively small, indicating that accurate ocean information alone is not enough to drive skill in this model. Instead, a coupled ocean or additional variables may be needed. We note that over the 1994-2016 period GenCast-Persisted achieves a correlation of 0.35 ($p=0.06$), lower than the value of 0.42 reported for ACE2 over the same period using 20 ensemble members (Kent et al. 2025). Unlike GenCast, ACE2 receives information about sea ice as an input, which could be a source of the skill difference between the two models.

Finally we investigate the reliability of probabilistic predictions of variables falling in the lower or upper terciles. Over all variables, GenCast-Persisted generally shows little skill compared to climatology. However, the probabilities for GenCast-Forced are well-calibrated compared to SEAS5, indicating that the missing variability in the sea surface temperature may be enough to produce well calibrated ensembles with GenCast.

We acknowledge several limitations of this study. Firstly, it is common with seasonal forecasts to perform hindcasts in order to correct biases and drifts in the forecasts. Since we have not performed this step for either the GenCast or SEAS5 forecasts, we cannot say to what extent differences in performance are related to different lead-time dependent biases, or which forecast would perform better if such biases were removed. We also note that a 30-year period may not be enough to fully understand the relative behaviours of these forecast models over all possible conditions, given the long term variability of the earth system; valuable future work would therefore be to extend our analysis to more decades. Finally, it is known that correlations for MSLP variability, such as the NAO, are very sensitive to the ensemble size (Dunstone et al. 2016), and SEAS5 typically shows higher correlations using 51 members as opposed to the 20 members selected here (Johnson et al. 2019). The comparison of NAO skill between GenCast and SEAS5 here, and the MSLP correlations more broadly, must therefore be viewed cautiously, and further analysis using a larger ensemble size would be required to draw robust conclusions.

Since the sea surface temperatures are prescribed, this is also not a demonstration of full seasonal forecasting, but an indication of how well GenCast has learned to model long-term dynamics having been trained at short timescales. In future work we intend to explore how coupling to a full dynamic or machine-learnt ocean model will change GenCast’s seasonal forecasting skill and reliability.

Overall, the results show promise in the use of generative models such as GenCast to perform seasonal forecasts, providing further validation as to how well the model has learnt to capture physical processes. It can reproduce the atmospheric response to drivers of variability on seasonal timescales, despite the limited role of these drivers on variability at the training timescale of 12 hours. The results motivate the further study of models similar to GenCast coupled with a dynamical or machine-learnt ocean model.

## Electronic Supplementary Material

Below is the link to the electronic supplementary material.

Supplementary Material 1

## Data Availability

The datasets used in the current study for initial conditions, boundary conditions, and baseline seasonal forecasts are available in the Copernicus Climate Change Service, Climate Data Store, (2023), for registered users. The following datasets were used: ERA5 hourly data on pressure levels from 1940 to present (DOI: https://doi.org/10.24381/cds.bd0915c6), ERA5 hourly data on single levels from 1940 to present (DOI: https://doi.org/10.24381/cds.adbb2d47), Seasonal forecast monthly statistics on pressure levels (DOI: https://doi.org/10.24381/cds.0b79e7c5), Seasonal forecast monthly statistics on single levels (DOI: https://doi.org/10.24381/cds.68dd14c3), Precipitation monthly and daily gridded data from 1979 to present derived from satellite measurement (DOI: https://doi.org/10.24381/cds.c14d9324). Accessed July-August 2025. The GenCast model code and weights are available via https://github.com/google-deepmind/graphcast.

## References

[CR1] Allen A, Markou S, Tebbutt W, Requeima J, Bruinsma WP, Andersson TR, Herzog M, Lane ND, Chantry M, Hosking JS, Turner RE (2025) End-to-end data-driven weather prediction. Nature 641(8065):1172–1179. 10.1038/s41586-025-08897-0 40112882 10.1038/s41586-025-08897-0 PMC12119340

[CR2] Behrangi A, Adler R, Huffman G, Bolvin D, Gu G, Nelkin E (2024) GpcpGPCP version 3.2 monthly satellite-gauge (sg SG) combined precipitation data set. NSF National Center for Atmospheric Research. Accessed July-August 2025. 10.5065/6A3J-P326

[CR3] Bi K, Xie L, Zhang H, Chen X, Gu X, Tian Q (2023) Accurate medium-range global weather forecasting with 3D neural networks. Nature 619(7970):533–538. 10.1038/s41586-023-06185-3 37407823 10.1038/s41586-023-06185-3 PMC10356604

[CR4] Christensen HM, Berner J (2019) From reliable weather forecasts to skilful climate response: a dynamical systems approach. Q J R Meteorol Soc 145(720):1052–1069. 10.1002/qj.3476

[CR5] Chen L, Zhong X, Li H, Wu J, Lu B, Chen D, Xie S-P, Wu L, Chao Q, Lin C, Hu Z, Qi Y (2024) A machine learning model that outperforms conventional global subseasonal forecast models. Nat Commun 15(1):6425. 10.1038/s41467-024-50714-1 39080287 10.1038/s41467-024-50714-1 PMC11289088

[CR6] Davey MK, Brookshaw A, Ineson S (2014) The probability of the impact of ENSO on precipitation and near-surface temperature. Climate Risk Manag 1:5–24. 10.1016/j.crm.2013.12.002

[CR7] Delaunay A, Christensen HM (2022) Interpretable deep learning for probabilistic MJO prediction. Geophys Res Lett 49(16):2022–098566. 10.1029/2022GL098566

[CR8] Dunstone N, Smith D, Scaife A, Hermanson L, Eade R, Robinson N, Andrews M, Knight J (2016) Skilful predictions of the winter North Atlantic Oscillation one year ahead. Nat Geosci 9(11):809–814. 10.1038/ngeo2824

[CR9] Hersbach H, Bell B, Berrisford P, Hirahara S, Horányi A, Muñoz-Sabater J, Nicolas J, Peubey C, Radu R, Schepers D (2020) The ERA5 global reanalysis. Q J R Meteorol Soc 146(730):1999–2049. 10.1002/qj.3803

[CR10] Ham Y-G, Kim J-H, Luo J-J (2019) Deep learning for multi-year ENSO forecasts. Nature 573(7775):568–572. 10.1038/s41586-019-1559-7 31534218 10.1038/s41586-019-1559-7

[CR11] Hurrell JW, Kushnir Y, Ottersen G, Visbeck M (2003) An overview of the North Atlantic Oscillation. North Atlant Oscillat Climat Signif Environ Impact 134:1–36. 10.1029/134GM01

[CR12] Hsu W-R, Murphy AH (1986) The attributes diagram a geometrical framework for assessing the quality of probability forecasts. Int J Forecast 2(3):285–293

[CR13] Johnson SJ, Stockdale TN, Ferranti L, Balmaseda MA, Molteni F, Magnusson L, Tietsche S, Decremer D, Weisheimer A, Balsamo G, Keeley SPE, Mogensen K, Zuo H, Monge-Sanz BM (2019) SEAS5: the new ECMWF seasonal forecast system. Geosci Model Develop 12(3):1087–1117. 10.5194/gmd-12-1087-2019

[CR14] Karlbauer M, Cresswell-Clay N, Durran DR, Moreno RA, Kurth T, Bonev B, Brenowitz N, Butz MV (2024) Advancing parsimonious deep learning weather prediction using the healpix mesh. J Adv Model Earth Syst 16(8):2023–004021. 10.1029/2023MS004021

[CR15] Kent C, Scaife AA, Dunstone NJ, Smith D, Hardiman SC, Dunstan T, Watt-Meyer O Skilful global seasonal predictions from a machine learning weather model trained on reanalysis data. npj Climate and Atmospheric Science 8:314 (2025). 10.1038/s41612-025-01198-3

[CR16] Kochkov D, Yuval J, Langmore I, Norgaard P, Smith J, Mooers G, Klöwer M, Lottes J, Rasp S, Düben P, Hatfield S, Battaglia P, Sanchez-Gonzalez A, Willson M, Brenner MP, Hoyer S (2024) Neural general circulation models for weather and climate. Nature 632(8027):1060–1066. 10.1038/s41586-024-07744-y 39039241 10.1038/s41586-024-07744-y PMC11357988

[CR17] Lang S, Alexe M, Clare MC, Roberts C, Adewoyin R, Bouallègue ZB, Chantry M, Dramsch J, Dueben PD, Hahner S (2024) AIFS-CRPS: ensemble forecasting using a model trained with a loss function based on the continuous ranked probability score. arXiv preprint: 2412.15832 10.48550/arXiv.2412.15832

[CR18] Ling F, Chen K, Wu J, Han T, Luo J-J, Ouyang W, Bai L (2024) FengWu-W2S: A deep learning model for seamless weather-to-subseasonal forecast of global atmosphere. arXiv preprint: 2411.10191 10.48550/arXiv.2411.10191

[CR19] Li G, Liu X, Cao S, Liang H, Chen M, Zhang L, Zhang J, Wang J, Jin M, Zheng J, Fu H (2025) TianQuan-Climate: A subseasonal-to-seasonal global Weather model via incorporate climatology state. arXiv preprint: 2504.09940. 10.48550/arXiv.2504.09940

[CR20] Lam R, Sanchez-Gonzalez A, Willson M, Wirnsberger P, Fortunato M, Alet F, Ravuri S, Ewalds T, Eaton-Rosen Z, Hu W, Merose A, Hoyer S, Holland G, Vinyals O, Stott J, Pritzel A, Mohamed S, Battaglia P (2023) Learning skillful medium-range global weather forecasting. Science. 10.1126/science.adi2336 10.1126/science.adi2336 37962497

[CR21] Liu Y, Zheng Z, Cheng J, Tsung F, Zhao D, Rong Y, Li J (2025) CirT: Global Subseasonal-to-Seasonal Forecasting with Geometry-inspired Transformer. arXiv preprint: 2502.19750. 10.48550/arXiv.2502.19750

[CR22] Mogensen K, Keeley S (2012) Coupling of the NEMO and IFS models in a single executable. Technical Meomorandum 673. https://www.ecmwf.int/en/elibrary/75709-coupling-nemo-and-ifs-models-single-executable. Accessed 2023-10-09

[CR23] McPhaden MJ, Zebiak SE, Glantz MH (2006) ENSO as an Integrating Concept in Earth Science. Science 314(5806):1740–1745. 10.1126/science.1132588 17170296 10.1126/science.1132588

[CR24] Nguyen T, Brandstetter J, Kapoor A, Gupta JK, Grover A (2023) ClimaX: A foundation model for weather and climate. arXiv preprint: 2301.10343 10.48550/arXiv.2301.10343

[CR25] Parthipan R, Anand M, Christensen HM, Vitart F, Wischik DJ, Zscheischler J (2025) Regularization of ML models for Earth systems by using longer model timesteps. arXiv preprint: 2303.18023. 10.48550/arXiv.2503.18023

[CR26] Palmer TN, Doblas-Reyes F, Weisheimer A, Rodwell MJ (2008) Toward seamless prediction: Calibration of climate change projections using seasonal forecasts. Bull Am Meteor Soc 89(4):459–470. 10.1175/BAMS-89-4-459

[CR27] Pinheiro E, Ouarda TBMJ (2025) An interpretable machine learning model for seasonal precipitation forecasting. Commun Earth Environ 6(1):1–14. 10.1038/s43247-025-02207-2 40125292 10.1038/s43247-025-02207-2 PMC11928313

[CR28] Price I, Sanchez-Gonzalez A, Alet F, Andersson TR, El-Kadi A, Masters D, Ewalds T, Stott J, Mohamed S, Battaglia P, Lam R, Willson M (2025) Probabilistic weather forecasting with machine learning. Nature 637(8044):84–90. 10.1038/s41586-024-08252-9 39633054 10.1038/s41586-024-08252-9 PMC11666454

[CR29] Siegert S, Bellprat O, Ménégoz M, Stephenson DB, Doblas-Reyes FJ (2017) Detecting improvements in forecast correlation skill: Statistical testing and power analysis. Mon Weather Rev 145(2):437–450. 10.1175/MWR-D-16-0037.1

[CR30] Scaife, A.A., Smith, D.: A signal-to-noise paradox in climate science. npj Climate and Atmospheric Science 1(1), 28 (2018) 10.1038/s41612-018-0038-4

[CR31] Von Storch H, Zwiers FW (1999) Statistical Analysis in Climate Research. Cambridge University Press, Cambridge 10.1017/CBO9780511612336

[CR32] Wilks DS (2011) Statistical Methods in the Atmospheric Sciences vol. 100. Elsevier. 10.1016/C2017-0-03921-6

[CR33] Weyn JA, Kumar D, Berman J, Kazmi N, Klocek S, Luferenko P, Thambiratnam K (2024) An ensemble of data-driven weather prediction models for operational sub-seasonal forecasting. arXiv preprint: 2403.15598. 10.48550/arXiv.2403.15598

[CR34] Watt-Meyer O, Henn B, McGibbon J, Clark SK, Kwa A, Perkins WA, Wu E, Harris L, Bretherton CS (2025) ACE2: accurately learning subseasonal to decadal atmospheric variability and forced responses. npj Climate Atmosph Sci 8(1):205. 10.1038/s41612-025-01090-0

[CR35] Zhou C, Chen L, Zhong X, Lu B, Li H, Wu L, Wu J, Hu J, Dou Z, Hsu P-C (2025) A machine learning model for skillful climate system prediction. arXiv preprint 2505.06269 10.48550/arXiv.2505.06269

[CR36] Zhao M, Held IM, Vecchi GA (2010) Retrospective forecasts of the hurricane season using a global atmospheric model assuming persistence of SST Anomalies. Mon Weather Rev 138:3858–3868. 10.1175/2010MWR3366.1

[CR37] ZhangG, Rao M, Yuval J, Zhao M Advancing seasonal prediction of tropical cyclone activity with a hybrid AI-physics climate model. Environmental Research Letters, 20:9 (2025). 10.1088/1748-9326/adf864

