# Supplementary material for: Seasonal forecasting using the GenCast probabilistic machine learning model
Source: Clim Dyn. 2026 Mar 16;64(4):148. doi: 10.1007/s00382-026-08077-4 (PMC12992418; doi:10.1007/s00382-026-08077-4)
Supplement: Supplementary file 1 — Supplementary Material 1 [file 382_2026_8077_MOESM1_ESM.pdf]

Supplementary Materials for: Seasonal forecasting using the GenCast probabilistic machine learning model

S1 Global Skill by Year

Figure S1 shows how the globally aggregated CRPS for each DJF seasonal prediction varies with initialisation year.

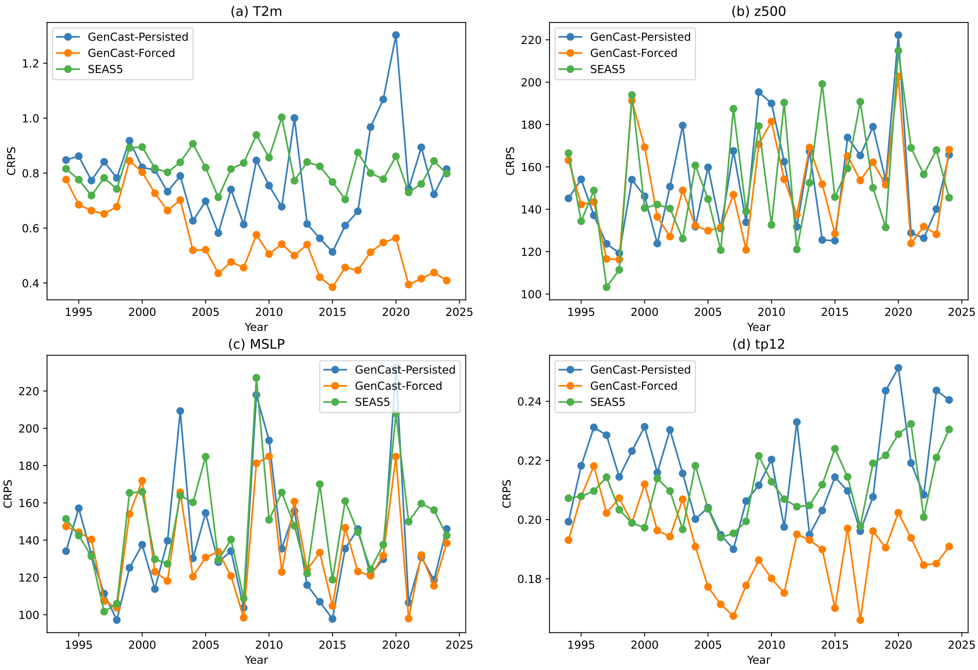

Fig. S1 Continuous Ranked Probability Score (CRPS) between the seasonal predictions and ERA5 reanalysis, aggregated globally for (a) 2-metre temperature (b) geopotential height at 500hPa (c) mean sea level pressure and (d) 12-hour precipitation.

S2 Skill by Month

Figure S2 shows the CRPS of the seasonal forecasts, averaged by forecast month.

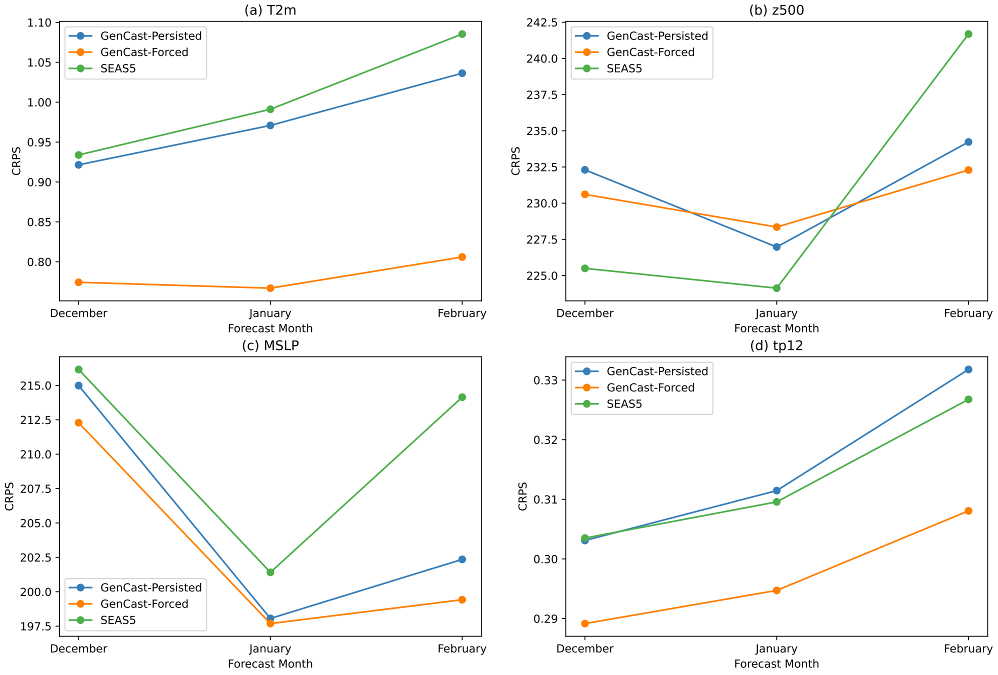

Fig. S2 CRPS between the seasonal predictions and ERA5 reanalysis, aggregated globally and and by forecast month (a) 2-metre temperature (b) geopotential height at 500hPa (c) mean sea level pressure and (d) 12-hour precipitation.

S3 Mean State Bias

Figures S3-S6 show the difference in mean seasonal prediction between forecasts and ERA5 reanalysis.

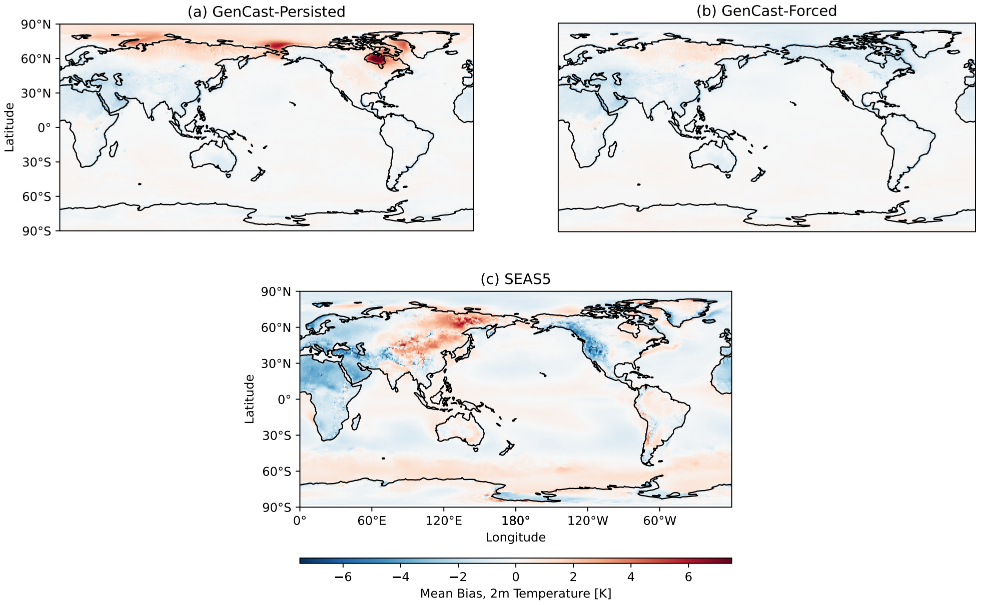

Fig. S3 Difference between the ensemble mean DJF seasonal forecasts of 2-metre temperature and the ERA5 reanalysis, for (a) GenCast-Persisted (b) GenCast-Forced and (c) SEAS5.

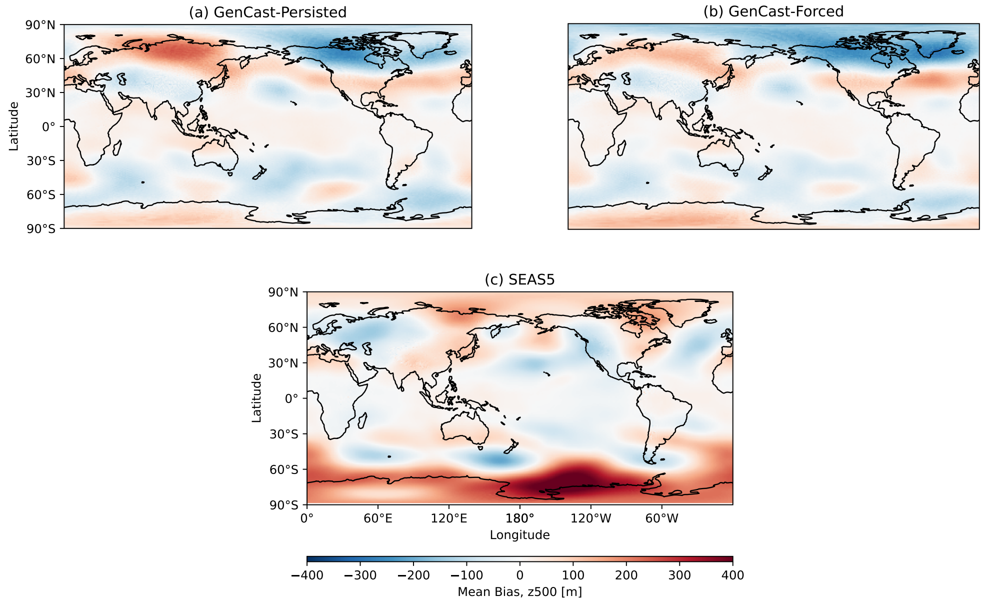

Fig. S4 As in Fig. S3 but for geopotential height at 500hPa.

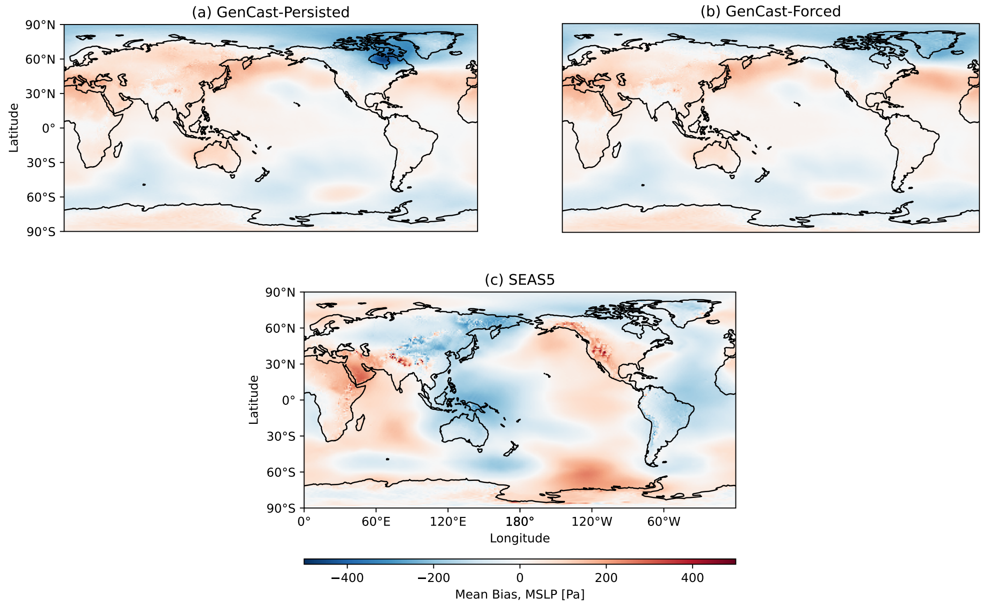

Fig. S5 As in Fig. S3 but for mean sea level pressure.

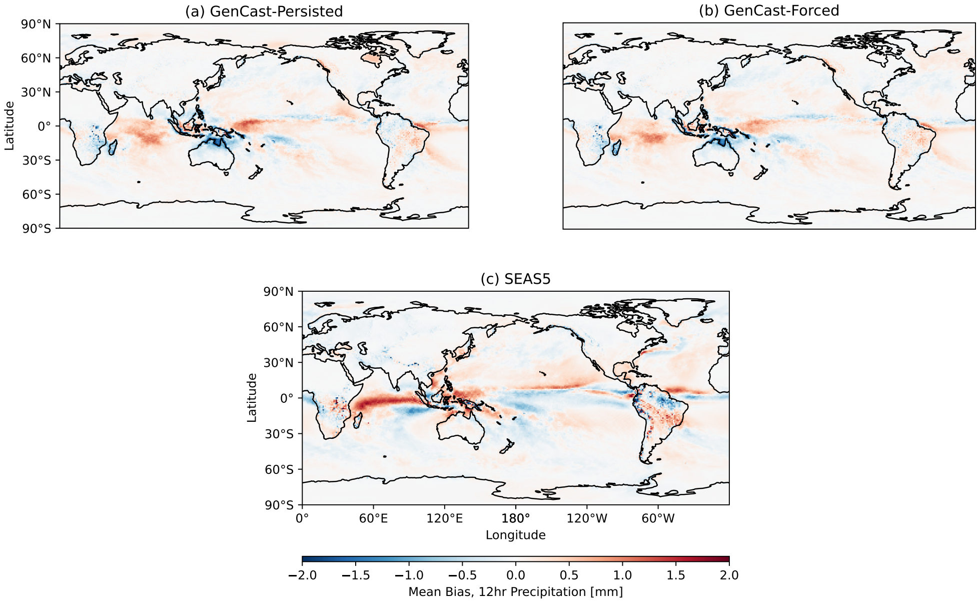

Fig. S6 As in Fig. S3 but for 12-hour precipitation.
